# Exploring non-linear effects of walking accessibility on well-being in rural older adults of Jintang County: a random forest analysis

**DOI:** 10.3389/fpubh.2024.1333510

**Published:** 2024-02-16

**Authors:** Haimei Li, Mingyang Li, Panyu Peng, Yi Long, Yibin Ao, Homa Bahmani

**Affiliations:** ^1^Humanities and Law School, Chengdu University of Technology, Chengdu, China; ^2^College of Management Science, Chengdu University of Technology, Chengdu, China; ^3^College of Environment and Civil Engineering, Chengdu University of Technology, Chengdu, China

**Keywords:** accessibility, well-being, random forest model, rural older adult, non-linear

## Abstract

**Objective:**

The global concern surrounding the aging population has brought the well-being of older individuals to the forefront of societal attention. Unfortunately, studies focusing on the well-being of older people residing in rural areas are frequently overshadowed by the developmental disparities between rural and urban regions. Thus, this study aims to delve into the non-linear impact of walking accessibility on the subjective well-being of rural older adults. The goal is to gain a comprehensive understanding of this relationship, ultimately contributing to an improved quality of life and health for older adults in rural areas.

**Methods:**

In this study, the Random Forest algorithm was employed to explore the non-linear effects of demographic variables, perceived safety, subjective built environment (including perceptions and preferences of the built environment), and walking accessibility on the subjective well-being of older adults.

**Results:**

The findings of this study underscore the pivotal role of walking accessibility in influencing the well-being of older adults, particularly in terms of access to bazaars and health centers, where non-linear and threshold effects are evident. Furthermore, community safety, road conditions, and walking preferences were identified as positive influencers on the well-being of older adults. Well-being trends varied with age, revealing noteworthy non-linear relationships for certain variables.

**Conclusion:**

The insights gained from this study provide crucial theoretical guidance for the development of policies tailored to the unique context of rural aging. By taking into account factors such as walking accessibility, community safety, health support, and social interaction, we can create an improved living environment for rural older adults, ultimately enhancing their happiness and overall quality of life.

## 1 Introduction

Contemporary social development and medical progress have led to lower fertility rates and increased life expectancy, contributing to the increasing prevalence of an aging population worldwide (1). The rural older adult population occupies a considerable proportion in many countries (2, 3). For example, in China, a country with the largest older adult population in the world, the rural older adult population over 65 years accounts for 18.57% of the rural population (4, 5). In the context of global aging, society is becoming more concerned about the well-being of older adults (6). The gap caused by the essential difference between urban and rural older adult care makes the subjective well-being research of the rural older adult population have practical significance and deserve people’s attention (7, 8). However, most studies on subjective well-being in older people do not focus on rural areas (9), and the well-being of rural older adults has received relatively little attention. Exploring the influence mechanism of the subjective well-being of the rural older adult population in China will provide a practical reference path for improving the flourishing of the global rural older adult population.

Subjective well-being refers to the cognitive evaluation of life satisfaction. As a comprehensive subjective feeling index, it can reflect older adults’ experience of life satisfaction and happiness (10). Although subjective well-being is a complex concept, empirical research has made great progress over the past decade (11–15). Existing literature has demonstrated that 30 to 40% of individuals’ well-being is attributable to genetic factors (13, 16). On the other hand, between 60 and 70% of subjective well-being can be attributed to environmental effects (16). Many studies have explored subjective well-being from multiple perspectives, including social environment, built environment, personal perception level, and so on (17–19). However, among these perspectives, mobility plays a significant role in maintaining the subjective well-being of older adults (17, 20–22). Low mobility may prevent older adults from engaging in social activities, leading to low mood, depression, and loneliness (23–26). A good living environment is a key factor in improving the mobility of older adults, which includes reasonable community planning, namely providing convenient walking roads and public facilities, and the creation of a safe and friendly living environment (27–30). Through these community planning measures, older people can be more willing to go out and travel by foot. In this context, walking accessibility becomes an essential factor affecting subjective well-being among older adults. Several studies have shown that walking accessibility directly affects residents’ subjective well-being (17, 19, 31). However, existing studies are mainly based on linear models, ignoring the complex and diverse non-linear influence relationships that may exist (29, 32, 33). Therefore, this study aimed to explore the non-linear effects of walking accessibility on subjective well-being in rural older adults. It is expected to help rural older adults improve their subjective well-being.

Because the built environment is considered to effectively affect the daily life of residents (34–36), its impacts on subjective well-being have always been one of the more comparatively noticed topics (4, 30, 37). As a special vulnerable group, older people’s physical health status and self-care ability will be more vulnerable to the built environment than other age groups (38), which might affect their subjective well-being (39). Generally, the built environment can be summarized as density, diversity, design, destination accessibility, and distance to transit (40). These elements are closely related to the vitality and attractiveness of the community, where destination accessibility directly affects the convenience of people to travel and thus affects the frequency of social interaction. If the destination’s accessibility is low, it may lead to spending more time and energy on travel, thus reducing social interaction and making people feel lonely (41). In addition, destination accessibility is also closely related to physical health and quality of life (42). A review suggests that environmental factors such as accessibility and green space quality can impact citizens’ physical activity (43). Destination accessibility can encourage people to walk, ride, or use public transport more often than relying on private cars, especially older people, who usually use cars less frequently (36). The present study fully considers the rural-specific environmental characteristics when exploring the subjective well-being of older people in rural areas. For example, rural areas may be more dispersed and distances between the walking target sites may be larger, which may affect walking accessibility and subjective well-being in older adults. At the same time, green and natural resources may be more abundant in rural areas, which will also affect the happiness of older adults. In addition to walking accessibility, the subjective well-being of rural older adults may be influenced by multiple other factors, for example, social support, health status, financial status, and community friendliness. Therefore, these potential confounding factors need to be controlled for or considered in studies to more accurately assess the effect of walking accessibility on subjective well-being in rural older adults. In conclusion, this study aimed to investigate the non-linear effects of walking accessibility on the subjective well-being of rural older adults and to consider the influence of rural environmental characteristics and other potential factors. Through scientific research design and rational statistical analysis, it is expected to provide helpful advice and decision support for improving the quality of life and subjective older adults’ well-being in rural regions, as well as for promoting sustainable development in rural communities.

This study conducted a detailed travel survey of 515 respondents in rural Sichuan, China, applying a random forest algorithm to explore the non-linear effects of demographic variables, safety perception, subjective built environment (perception and preference), and walking accessibility on the subjective well-being of older adults. Compared with traditional linear models, random forest is better able to estimate the relative importance of different influencing factors to the dependent variable and effectively capture the non-linear relationship between features, thus providing more accurate and reliable prediction results. The study’s contribution encompasses three aspects: firstly, it re-examined the influence of walking accessibility, subjective building environment, and safety perception on older adults, emphasizing the importance of these factors in affecting older adults’ well-being. Secondly, this study provides a new perspective on rural construction layout planning for the well-being improvement of older adults in rural areas. Third, machine learning was used to explore the nonlinear relationships, providing empirical support and valuable reference for improving the life happiness of older people residing in rural.

## 2 Method and data

### 2.1 Study area, survey method, and data

Jintang County, affiliated with Chengdu City, Sichuan Province, is located northeast of the Chengdu Plain. By the end of 2021, the total registered population of Jintang County had reached 904,000 (44). As an important link to the Chengdu Plain economic circle, Jintang County has been identified as the characteristic industrial development area of Chengdu City and is a typical demonstration county of urbanization development in Chengdu suburbs (45). Therefore, in this study, under the background of Jintang County, Guancang and Qingjiang towns were randomly selected from 19 towns/streets as samples, and on this basis, 11 sample villages were selected for household survey in January 2021. The following points are mainly considered when determining the sample villages: First, the rural transportation and infrastructure are well-developed. Second, Guancang Town and Qingjiang Town residents have a relatively traditional lifestyle, and the pace of life is relatively slow. Most farmers still take farming as their main occupation, and some also engage in aquaculture, which aligns with the lifestyle of Chinese rural residents.

**Figure 1:**
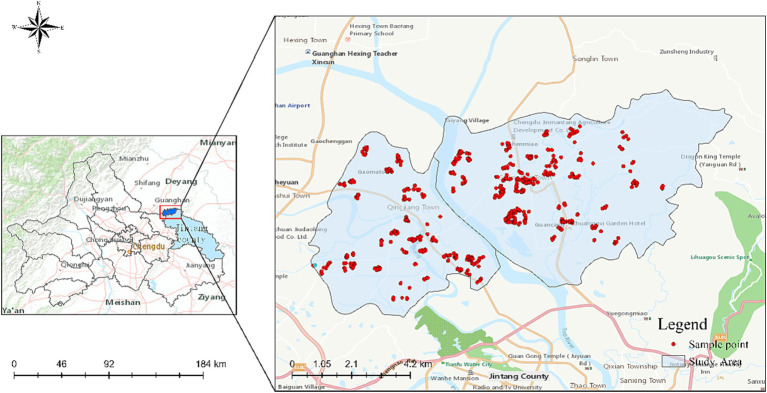
Sample distribution map.

The data used in this study are mainly in two parts. First, individual-level data includes socio-demographic information, subjective well-being measures, built environment perception and preferences, and physical activity information for older adults (see Appendix for descriptions of specific variables). In the process of the investigation, a random one-to-one questionnaire survey was conducted, ensuring that every individual in the target population had an equal chance of being selected. Researchers approached every older adult encountered in the survey area. If a respondent was unwilling to participate, the researcher would promptly move on to the next individual. This approach maximized the reduction of any potential subjectivity or selection bias from the researchers’ side. Each questionnaire took an average of 9 min to complete. In total, 545 questionnaires were recovered, of which 30 were excluded due to incomplete information or incorrect filling, and 515 were valid questionnaires, with a recovery rate of 94.50% (The sample distribution is shown in Figure 1). The other part of the data concerns built-up environmental accessibility, which researchers on the spot measured. The researchers used the Ovitalmap to measure the distance from the sample households to the market, health center, bus station, main road, older adult activity center, square (park), supermarket (canteen), village committee, kindergarten, primary school, town center and other destinations in the sample village.

### 2.2 Variables

The dependent variable was subjective well-being, using the Memorial University of Newfoundland Scale of Happiness (MUNSH). MUNSH is a commonly used self-rating scale of the subjective well-being of older adults, developed by Kozma and Stones (46). MUNSH has 24 items reflecting positive emotion (PA), negative emotion (NA), positive experience (PE), and negative experience (NE). The final score of the SWB was calculated. The calculation formula is as follows:


SWB=PA+PE−NA−NE

Independent variables included walk accessibility, socio-demographic information, built environment perception and preferences, and physical activity information. The demographic variables mainly included gender, age, marital status, education level, resident population status, and health status. Among the respondents, 194 (37.7%) were male and 321 (62.3%) were women; 121 (23.6%) aged 60–65 years, 143 (27.8%) aged 66–70 years, 103 (20.0%) aged 71–75 years, 70 (14.1%) aged 76–80 years, 66 (12.8%) aged 81–90 years, and 8 (1.7%) aged over 90 years. The demographic information of the respondents is shown in Table 1. Subjectively perceived variables were road conditions, safety near the residence, travel convenience, and walking preference. Road conditions were measured by six items, safety near residence was measured by seven items, four items measured travel convenience conditions, and walking preference was measured by one item (See the attached table for the specific question items). The five-point Likert scale defines all the items. Walking accessibility in this study was measured on-site by researchers using the Ovitalmap. The data calculation process referred to a study by Ao et al. (47). The calculation formula is as follows:


Accessibility=1dn+1

**Table 1:** Demographic information.

Population basic information category	Variable type	Variable-definition		Amount	Percent (%)
Gender	Classified variable	Male	Gender1	194	37.7
		Female	Gender2	321	62.3
Age	Sequence variable	60–65	Age1	121	23.6
		66–70	Age2	143	27.8
		71–75	Age3	103	20.0
		76–80	Age4	70	14.1
		81–90	Age5	66	12.8
		>90	Age6	8	1.7
Education	Sequence variable	No	Education1	228	44.3
		Elementary school	Education2	229	44.5
		Junior middle school	Education3	48	9.2
		Senior middle school	Education4	8	1.6
		University and above	Education5	2	0.4
Marriage	Classified variable	Married	Marriage1	412	80.0
		Unmarried	Marriage2	102	20.0
Physical condition	Sequence variable	Good health	Health1	45	8.7
		Relative health	Health2	263	51.1
		Normal health	Health3	123	23.9
		Bad health	Health4	72	14.0
		Worse health	Health5	12	2.3

### 2.3 Method

**Figure 2:**
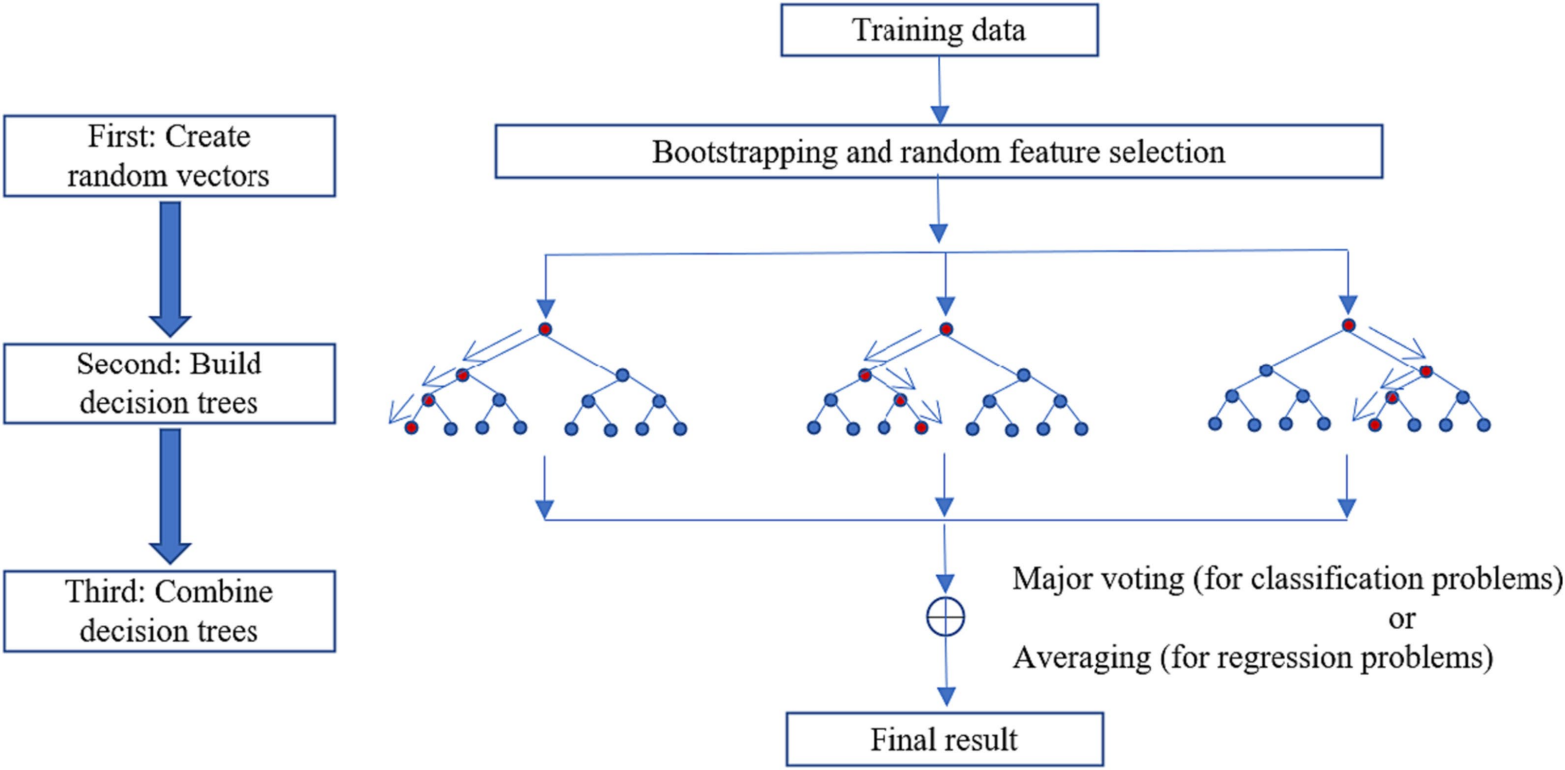
Schematic representation of the random forest.

The random forest (RF) model was used to explore the non-linear relationships between socio-demographic factors, subjective perception, walking accessibility, walking accessibility in daily behaviors, and older adults’ subjective well-being in rural areas. The random forest model is a machine-learning algorithm based on an integrated learning framework. It works by creating multiple decision trees and making predictions based on the majority votes of all single trees, as is shown in Figure 2. Random forest has been widely used in constructing the environment, transportation, and other fields (29, 36, 48). Compared with traditional linear models, the random forest has better predictive performance on model selection, suitable for different types of explanatory variables (continuous and discrete), and has good flexibility, generalization, and resistance to overfitting.

However, the random forest model is inferior to the conventional linear model in terms of interpretability, known as the black-box problem in the field of machine learning. To address this problem, various methods of interpreting machine learning have emerged, such as locally interpretable model-agnostic interpretation (LIME), cumulative local effects (ALE), partial correlation maps (PDP), and SHAP. In this study, we introduced the PDP method for visualizing the nonlinear relationships and importance assessment between the explanatory and dependent variables. The PDP explains the model by demonstrating the marginal effect of the feature variable on the dependent variable, avoiding reliance on *a priori* assumptions. We will use the PDP to demonstrate the nonlinear relationship of independent variables on subjective well-being in rural older adults (49, 50).


$$\hat{\phi }\left(X_{1}\right)=\frac{1}{T}\overset{T}{\underset{i=1}{\sum }}f\left(X_{1},X_{i2},\ldots ,X_{ij}\right)$$

In the formula, the dependent variable is $f\left(x\right)$ ; $\hat{\phi }\left(X_{1}\right)$ is the partial correlation function of the feature variable $X_{1}$ ; T represents the number of instances in the data set; $X_{1},X_{i2},\ldots ,X_{ij}$ is the actual observation of the j th feature of sample i.

In conclusion, this study will use random forest models and explanatory machine learning methods to deeply explore the socio-demographic factors, subjective perception, walking accessibility, and the nonlinear relationship and characteristic importance assessment of daily behavioral walking accessibility and the subjective well-being of rural older adults. By applying this method, this study will provide helpful information for understanding rural older adult travel and provide scientific support for improving travel planning and neighborhood environment design.

In the process of optimizing the random forest model, three core parameters are mainly adjusted: the maximum tree depth, the number of features of a single tree, and the total number of trees. A grid search technique is introduced to find out the optimal combination of these parameters, and arbitrary settings of the parameters are avoided (51). The grid search process is as follows: first, set the parameter range, the maximum depth of the largest tree is 1–20, the number of single tree features is 2–10, and the number of trees is 10–1,000 (interval 10). Then, all 18,000 (=20 × 9 × 100) possible parameter combinations were evaluated to test the model performance by out-of-bag error (52). After 18,000 tests, it was found that the model showed the best performance when the maximum depth was set to 6, the number of features was set to 9, and the total number of trees was set to 210. Therefore, this parameter combination was chosen as the optimal model, and a subsequent analysis was performed.

## 3 Results

### 3.1 Relative importance of the independent variables

**Figure 3:**
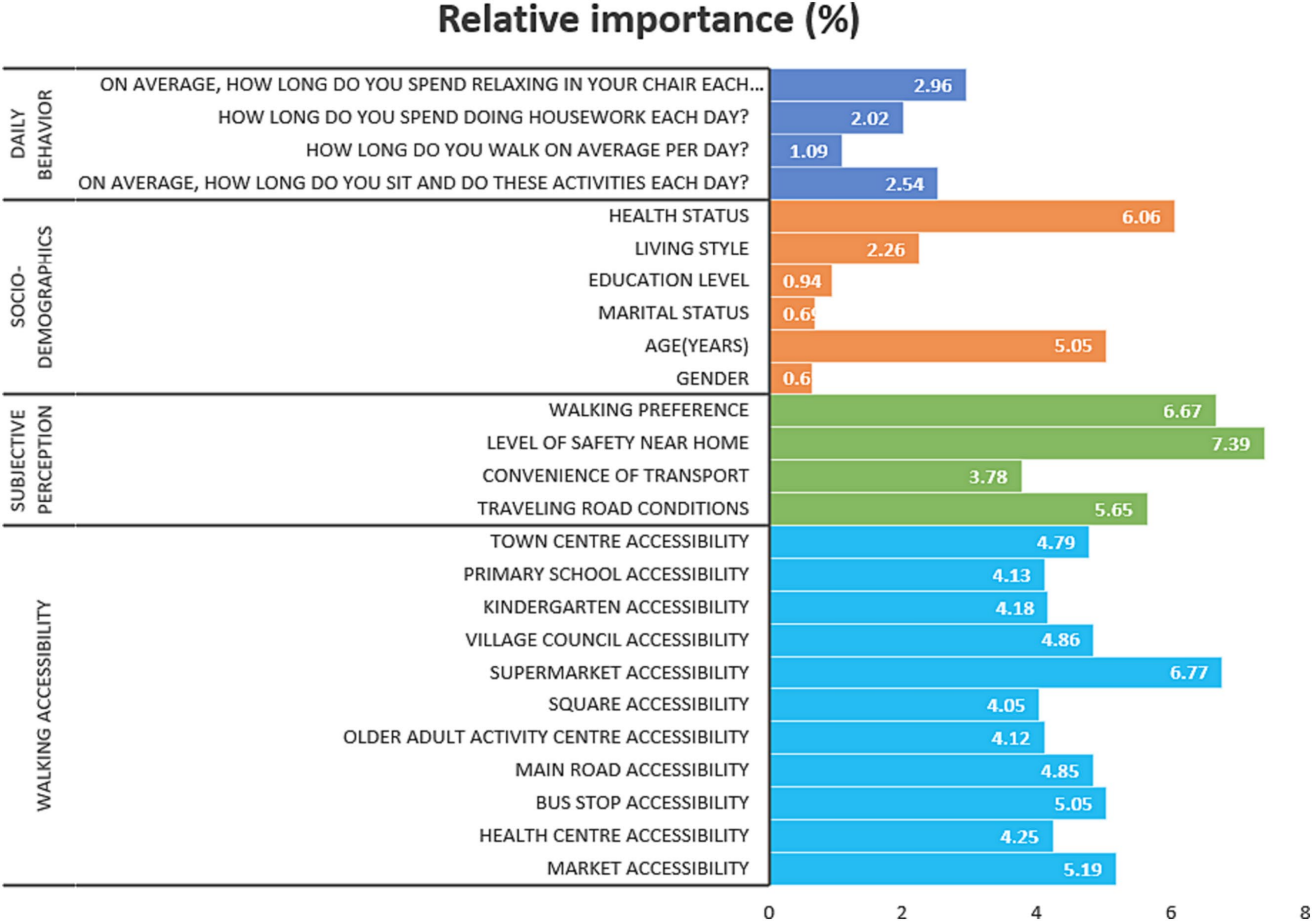
Relative importance.

Table 2 and Figure 3 shows the relative importance of the independent variables in predicting subjective well-being among rural older adults; the higher the relative importance, the more significant the ability of the independent variable in the prediction. Notably, walking accessibility had the highest overall contribution of all the independent variables. This finding not only confirms previous research findings but also highlights the importance of walking accessibility in the subjective well-being of older adults in rural areas (36, 53, 54).

**Table 2:** Relative materiality.

	**Variable**	**Relative importance (%)**	**Total (%)**
Walking accessibility	Market Accessibility	5.19	52.25
	Health Centre Accessibility	4.25	
	Bus Stop Accessibility	5.05	
	Main Road Accessibility	4.85	
	Older adult Activity Centre Accessibility	4.12	
	Square Accessibility	4.05	
	Supermarket Accessibility	6.77	
	Village Council Accessibility	4.86	
	Kindergarten Accessibility	4.18	
	Primary School Accessibility	4.13	
	Town Centre Accessibility	4.79	
Subjective perception	Traveling road conditions	5.65	23.49
	Convenience of transport	3.78	
	Level of safety near home	7.39	
	Walking preference	6.67	
Socio-demographics	Gender	0.65	15.65
	Age(years)	5.05	
	Marital Status	0.69	
	Education level	0.94	
	Living style	2.26	
	Health Status	6.06	
Daily behavior	On average, how long do you sit and do these activities each day?	2.54	8.61
	How long do you walk on average per day?	1.09	
	How long do you spend doing housework each day?	2.02	
	On average, how long do you spend relaxing in your chair each day?	2.96	
**Total relative importance**			100

Furthermore, subjective perceived variables were studied in depth, focusing on the effect of safety of residence and other factors on subjective well-being among rural older adults. In terms of subjective perceived variables, the safety of residence was identified as the most critical factor in predicting subjective happiness, with a contribution rate as high as 7.39%, which highlights the vital importance of community security for the life satisfaction of older people, because, in rural areas, community safety conditions may affect the social activities, travel habits, and overall quality of life of older adults. On the other hand, in the socio-demographics category, the relative importance of health status was relatively high, reaching 6.06%, and that of age also reached 5.05%. This finding suggests that health status and age play an essential role in the subjective well-being of rural older adults. It is worth mentioning that the relative importance of marital status, education level, and gender was low, all contributing less than 1%. Perhaps in rural areas, these factors have relatively weak effects on older adults subjective well-being.

### 3.2 Non-linear effects of the independent variables on the SWB

**Figure 4:**
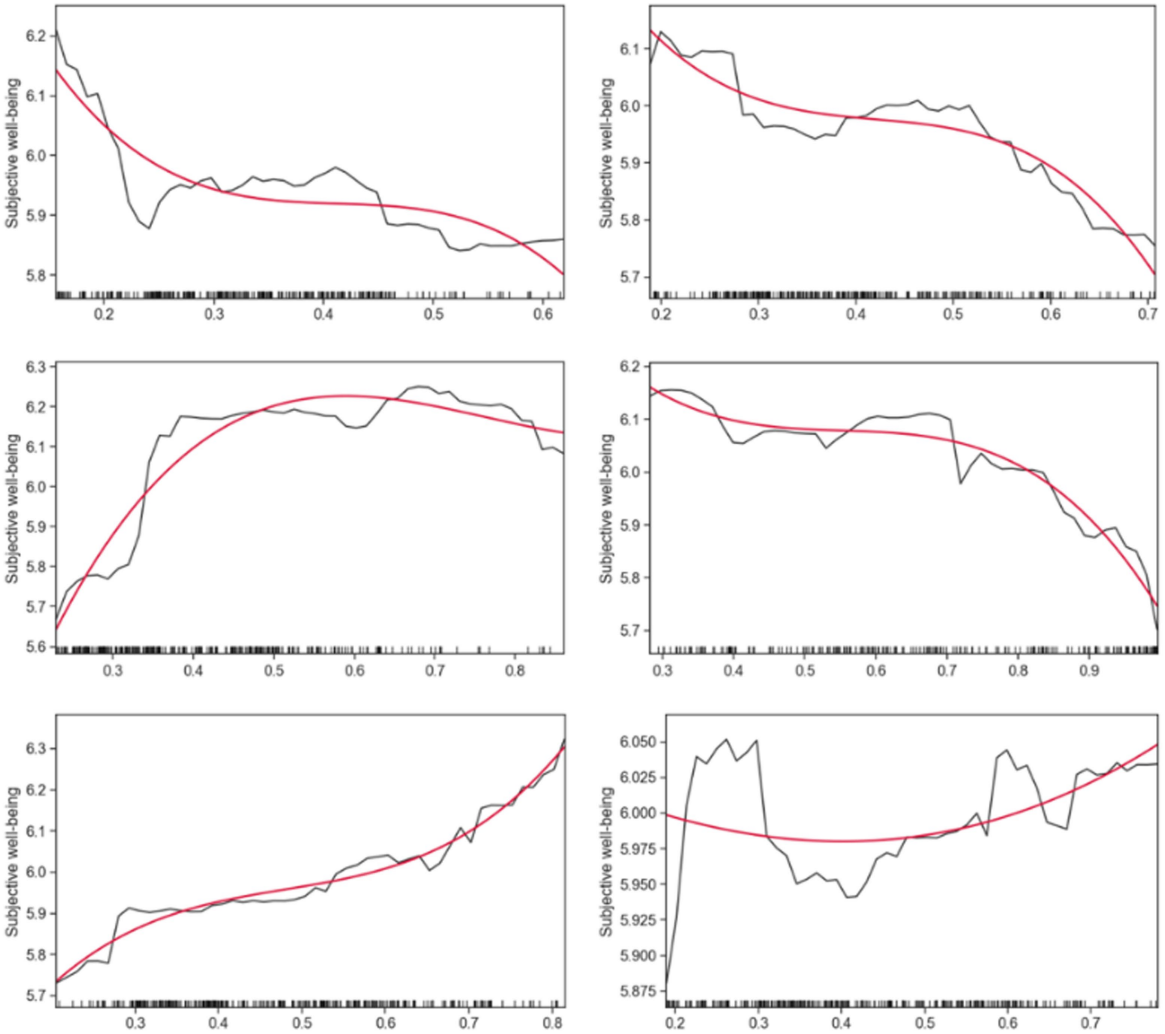
Nonlinear effects of walking accessibility on subjective well-being.

**Figure 5:**
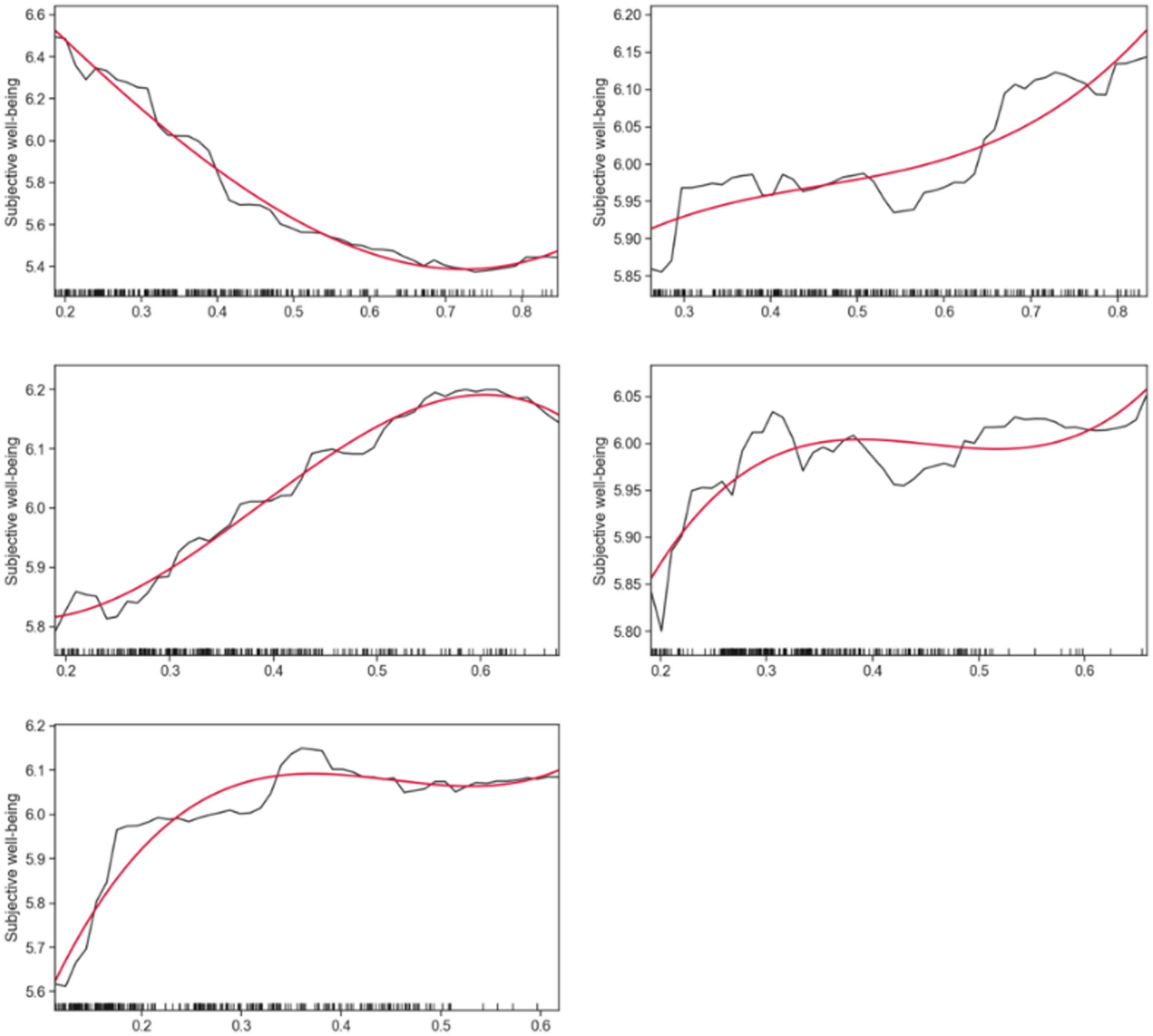
Nonlinear effects of walking accessibility on subjective well-being.

**Figure 6:**
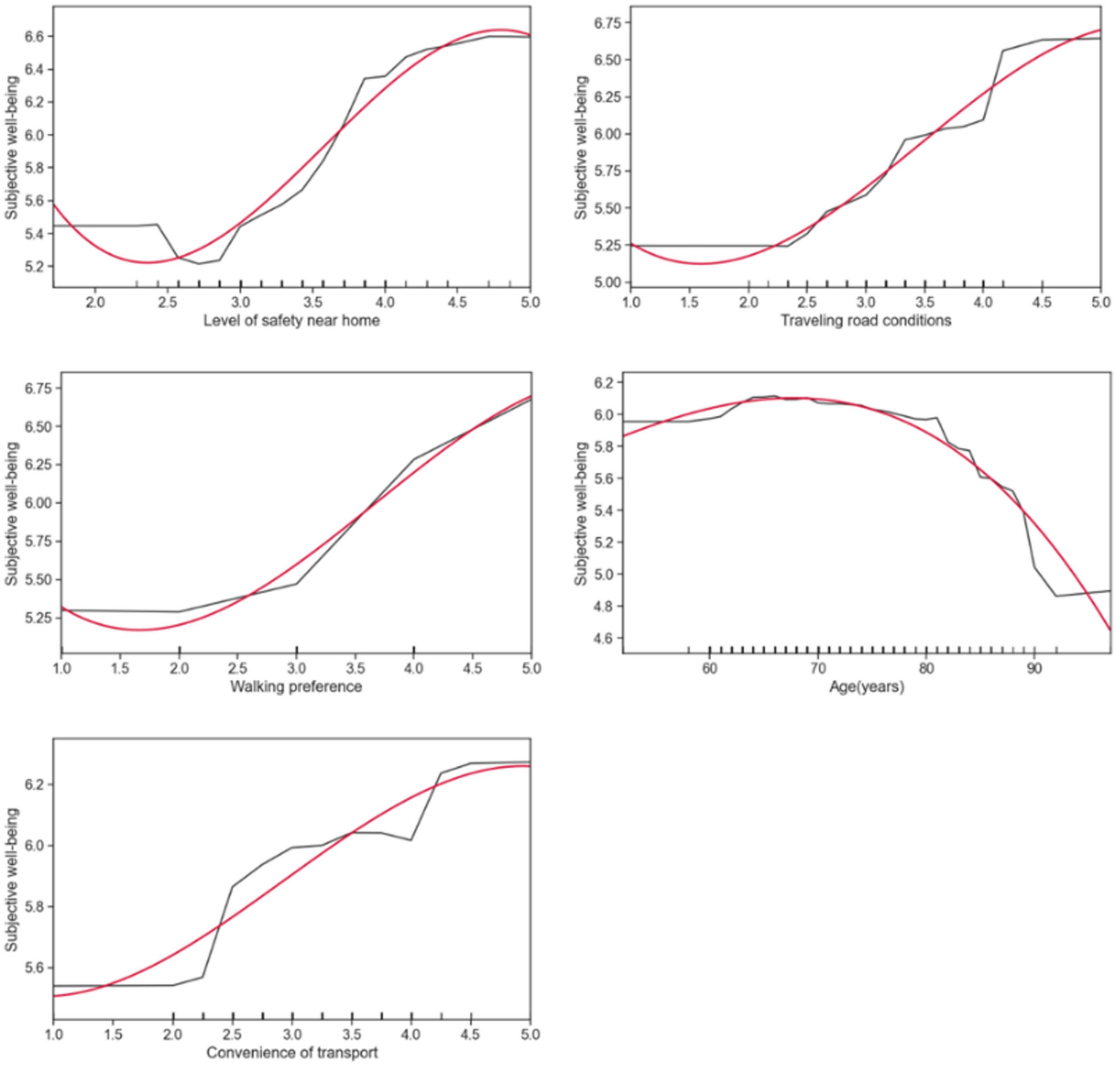
Nonlinear effects of walking accessibility on subjective well-being.

In this section, random forests are used to generate partial correlation maps (PDP) to more intuitively demonstrate the non-linear relationship between walking accessibility and the subjective well-being of rural older people. Figures 4–6 show the partial dependency plots generated for each variable of the random forest model, with the *x*-axis representing the distribution of independent variables and the *y*-axis representing the subjective well-being of rural older adults. To reduce noise and better describe the relationship between the independent and dependent variables, smooth curves were drawn using the Scipy library in Python (55). The data from the partial dependency plots showed that most walking accessibility factors showed non-linear and threshold effects in terms of subjective well-being in rural older adults.

Figures 4, 5 show that rural older adults’ subjective happiness initially decreases with increasing market accessibility. However, when accessibility reached 0.2411, happiness began to plateau and remained relatively flat. However, once the market accessibility exceeded 0.4107, the subjective happiness of older adults began to decline again. This trend is also equal between health center accessibility and subjective well-being.

As for the accessibility of destinations such as older adult activity centers, village committees, primary schools, and town centers, the trend is quite consistent. As the accessibility in these places increases, the subjective happiness of the rural older adults gradually increases, then stabilizes within a certain range, and then rises again. In contrast, the effect of accessibility at bus stops and kindergartens on subjective happiness showed a pattern of increasing first and then stabilizing. In particular, it is noteworthy that kindergarten accessibility has a significant threshold effect on subjective well-being, which will reach the threshold once the accessibility reaches 0.5957.

Furthermore, plaza accessibility increased within the accessibility from 0.1890 to 0.2976, leading to a rapid rise in subjective happiness. However, once accessibility exceeded 0.2976, subjective happiness began to decline. Similarly, supermarket accessibility showed a similar trend, with happiness gradually decreasing with increasing accessibility, reaching a threshold at 0.7376, after which happiness stabilized. The accessibility of the main road was relatively stable in the range of 0.2821 to 0.7044. However, subjective happiness began to decline sharply when trunk road accessibility exceeded 0.7044.

Figure 6 illustrates key variables associated with subjective well-being in rural older adults and other influencing factors besides walking accessibility. The clear nonlinear relationship between these critical variables and subjective well-being in rural older adults corresponds with the original study’s expectations. It can be observed from the partial dependence chart that the key variables such as safety, road conditions, walking preference, and travel convenience all have a positive impact on the subjective happiness of older people in rural areas with the increase of independent variables. This trend implies that older adults’ subjective well-being increases as these factors improve. However, it is worth noting that different relationships exist between age and subjective well-being in rural older adults. Between the ages of 50 and 69, the trend in older adult subjective happiness increased slowly but remained relatively stable. However, after reaching age 69, subjective well-being began to decline gradually, suggesting a threshold effect at some age stage, after which subjective well-being gradually decreases with age.

## 4 Discussion

To have a deeper understanding of the factors that affect the happiness of older people in rural areas to improve their quality of life, we chose Jintang County of Chengdu City as the research object to carry out research in 11 sample villages. We explored the nonlinear relationship between walking accessibility and subjective well-being in rural older adults. Using the random forest model, the following conclusions were gained:

First, walking accessibility had the highest overall importance for the subjective happiness of rural older adults, reaching 52.25%. The walking accessibility of different destinations has different effects on the subjective happiness of rural older adults, as Xu et al. (56), with consistent results. With the improvement of market and health center accessibility, the subjective happiness of rural older adults will show a decline, stable, and then decline. This trend can be explained in terms of real-life conditions and psychological factors. When the market accessibility increases, it may bring some adverse effects. As known from the research by Delbosc (57), such as traffic congestion, noise, and so on, which may make them feel inconvenient and dissatisfied, thus reducing their happiness. Similarly, accessibility to health centers may show a similar trend, with initial improvements that may mean better healthcare access, but excessive accessibility may not further increase happiness and even cause excessive worry or discomfort. These conditions may explain why, within a specific range, increased accessibility improves well-being, but either too high or too low may lead to decreased well-being. This finding also highlights the importance of balancing various factors to enhance the happiness of older people. In conclusion, some suggestions can be made for rural areas to help improve the subjective well-being of older people in rural regions. First, attention should be paid to improving walking accessibility to provide more convenient travel conditions, thus increasing the opportunities for interaction and activities between older people and the community. Secondly, strengthen the safety measures in the community, improve the sense of security of older adults, and create a comfortable and secure living environment for them. In addition, focusing on the health status of older adults and providing health care and health services can help to improve their quality of life and happiness. Finally, given the impact of age on subjective happiness, society can meet their needs by holding activities suitable for different age groups to improve their overall satisfaction. These suggestions take into account the reality and needs of older adults in rural areas and improve subjective well-being by improving their living environment, security, health status, and social activities.

In terms of accessibility to destinations such as older adult activity centers, village committees, primary schools, and town centers, the trend of subjective well-being is relatively consistent and generally gradually increased. This finding echoes the research of Lättman et al. (58), which indicates that accessibility is related to both travel satisfaction and life satisfaction among the older adults. Sites such as older adult activity centers, village committees, and town centers often provide opportunities for social interaction and cultural activities, so with increasing accessibility, the rural older adult may be more likely to participate in these community activities. According to Onishi et al. (59), this participation enhances social support and interaction, thereby enhancing well-being. Within a certain range, the increased accessibility makes it easier for older adults to integrate into community life and enjoy more social support and interaction. Increased accessibility at bus stops may mean that it is easier for older people to travel to different destinations, increasing their range of activities and social opportunities and enhancing happiness. The study by Zhu et al. (60) reflects that the most crucial factor affecting the well-being of older adults is the distance from home to high school. Regarding the accessibility of kindergartens and primary schools, its impact on happiness might be linked to the role of older adults within the family. Older adults play a crucial role in cultural inheritance and the structure of the family (61). Therefore, convenient access to schools enables them to more easily visit their grandchildren or participate in school activities, which could enhance their sense of happiness. However, the noise and crowding around schools might negatively affect some older adults. For square accessibility, the rapid rise in happiness may be because these sites often provide a social gathering place or shopping center, allowing rural older adult to participate in social activities or shopping, thus enhancing their emotional satisfaction. However, once accessibility exceeds a certain degree, it may lead to excessive crowding and noise, thus reducing well-being.

Finally, the key variables such as safety near home, road conditions, walking preference, and travel convenience positively impact the subjective happiness of older adults in rural areas with the increase of independent variables. Probably because they are directly related to the quality of life and everyday experiences of older people. Safer and more convenient roads can reduce older people’s travel pressure and insecurity (62). Similarly, increased walking preferences may enhance social interaction and daily activities, enhancing well-being. The different relationship between age and subjective happiness may be because older people at different age stages face different life needs and psychological states. Older people aged 50 to 69 are likely to be more active, with more social interactions and daily activities, leading to a more positive sense of well-being compared to those aged 70 and over. However, a threshold effect at age 69 may occur, possibly because a gradual decline in physical condition and decreased social circles affect well-being. Aging with increasing physical health and social interaction limitations may lead to a progressive decrease in subjective well-being (63).

In conclusion, this study analyzed the relationship between walking accessibility and the subjective well-being of rural older adults using a random forest model by investigating Jintang County in Chengdu. The study found that walking accessibility had an important impact on well-being, and accessibility at different destinations had different effects on happiness. In addition, factors such as residence safety, travel road conditions, walking preference, and travel convenience also positively impact the subjective happiness of rural older adults. There are differences in the effects of age on well-being, and increasing age may lead to a gradual decrease in well-being. These findings provide important references and enlightenment for improving the quality of life of rural older adults.

## 5 Conclusion

This study investigated the relationship between walking accessibility and subjective well-being in rural older adults. The results show that walking accessibility plays a vital role in affecting the happiness of older adults, especially the accessibility of markets and health centers, which offers non-linear and threshold effects. At the same time, community safety, travel road conditions, walking preference, and other factors also positively impact happiness. Age and age differ in happiness trends, with some variables showing clear nonlinear relationships. Based on the above research content and results, at the same time, the rural situation and the older adult living habits, the study puts forward the following suggestions to improve the quality of rural older adult life and happiness and help the government, community, and related agencies to take targeted measures to meet the needs of older adults, improve their life satisfaction and happiness:

1. Improve walking convenience: In rural areas, the development of public transportation may be limited, so improving walking convenience is a feasible approach. The government can invest in improving the roads and sidewalks in the village to ensure that it is easy for older adults to go to markets, health centers, and other places. At the same time, resources in rural areas are relatively limited, so it can be considered to build multi-functional activity places, such as activity centers for the older adult, for the older adult to have leisure, social, and fitness activities to enhance happiness further.
2. Strengthening community policing and patrols: security issues may affect the well-being of older adults. Strengthening community patrols and policing to improve the sense of security in the community can make older people go out more at ease. Moreover, interpersonal relationships are closer in rural communities. Encouraging older adults to participate in community activities and cultural inheritance and enhancing community cohesion will help to improve their happiness.
3. Focus on health promotion and support: Due to the limited medical resources, the government and the community can strengthen health promotion, provide basic health knowledge, and encourage older adults to adopt a healthy lifestyle.

These recommendations are based on realities and fully consider resource constraints and characteristics in rural areas to ensure the rationality and feasibility of implementation. By considering walking accessibility, community safety, health support, and social interaction, we can create a better living environment for rural older adults and improve their happiness and quality of life.

## Data availability statement

The raw data supporting the conclusions of this article will be made available by the authors, without undue reservation.

## Ethics statement

Ethical review and approval were not required for the study involving human participants in accordance with the local legislation and institutional requirements. The patients/ participants provided their written informed consent to participate in this study.

## Author contributions

HL: Conceptualization, Funding acquisition, Investigation, Project administration, Resources, Software, Validation, Writing – review & editing. ML: Conceptualization, Data curation, Investigation, Methodology, Software, Visualization, Writing – original draft, Writing – review & editing. PP: Conceptualization, Data curation, Formal analysis, Investigation, Methodology, Project administration, Software, Visualization, Writing – original draft, Writing – review & editing. YL: Methodology, Software, Visualization, Writing – original draft. YA: Conceptualization, Funding acquisition, Methodology, Resources, Supervision, Validation, Writing – review & editing. HB: Writing – review & editing.

## Appendix

**TABLE A** Variable description.

**Table:**

Travel road conditions	Travel has very good sidewalks.
	Travel has very nice bike lanes.
	Travel has a very good motorway.
	Convenient access to the public transit station.
	Travel to the destination is convenient and direct.
	Public facilities maintenance and service is very good.
Security degree near the place of residence	The trip process is very safe.
	The road to travel is broad.
	The road is smooth.
	There is no burden during the trip.
	There were no traffic accidents near my home.
	Daily necessities can be bought over a short range.
	No crime has occurred near the residence.
Travel convenience	Motorcycle riding is fast and convenient.
	Cycling is fast and easy.
	It is convenient and easy to take a travel tricycle.
	Other modes of travel (except bus, battery car, motorcycles, bicycles, walking and tricycles) are convenient and fast.
Walking preference	I like walking.
